# Beyond resistance: Emerging methods to dissect drug responses in *Leishmania*

**DOI:** 10.1371/journal.ppat.1014331

**Published:** 2026-06-24

**Authors:** Beatriz Cristina Dias de Oliveira, Jorge Arias del Angel, Nicole Herrmann May, Michael P. Barrett, Malgorzata Anna Domagalska, Tom Beneke

**Affiliations:** 1 Department of Cell and Developmental Biology, Biocenter, University of Würzburg, Würzburg, Germany; 2 School of Infection & Immunity, College of Medical, Veterinary and Life Sciences, University of Glasgow, Glasgow, United Kingdom; 3 Experimental Parasitology Unit, Department of Biomedical Sciences, Institute of Tropical Medicine, Antwerp, Belgium; University of Ostrava: Ostravska univerzita, CZECHIA

## Abstract

Treatment failure and relapse remain major challenges in leishmaniasis despite available chemotherapies. Historically, these outcomes have been interpreted through the lens of classical drug resistance driven by heritable genetic mutations. However, drug responses are increasingly recognised to extend beyond resistance and include distinct but related phenomena such as hypersensitivity, tolerance, and persistence. Dissecting this range of responses in *Leishmania* requires approaches that capture both heritable genetic variation and dynamic cellular states. Here, we highlight how emerging genomics and perturb-omics technologies can resolve mechanisms underlying diverse drug responses. We emphasise that their impact depends on state-aware experimental design, including calibrated drug-selection, varied exposure regimens, and strategies to isolate rare persister populations. Together, these approaches provide a framework to move beyond resistance-centric models towards a more comprehensive understanding of parasite drug response.

## Why the range of drug response diversity matters

**Fig 1:**
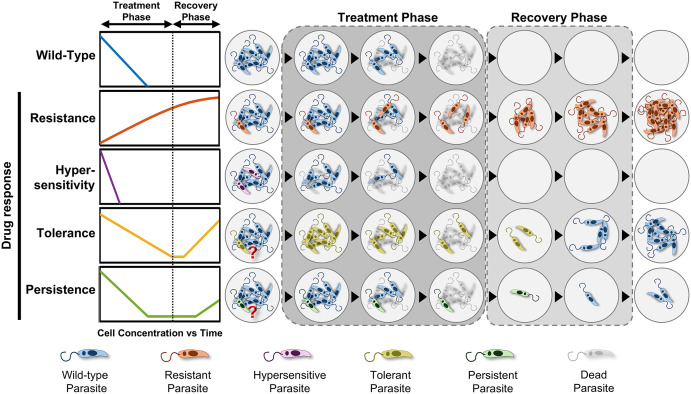
Phenotypic responses to drug treatment in *Leishmania.* Simulated population trajectories during drug exposure and recovery resolve five distinct drug response outcomes: wild-type (blue), resistant (orange), hypersensitive (purple), tolerant (yellow), and persistent (green). A corresponding schematic summarises these phenotypes, with rows indicating response classes and columns representing temporal progression from pre-treatment baseline through treatment (dark grey), recovery (light grey), and final post-recovery states. Colours are consistent between panels. Drug resistance is characterised by sustained growth despite drug exposure. In contrast, hypersensitivity represents the opposite phenotype, resulting in mutants that decline in population size faster than the wild-type. Persistence and tolerance are associated with a dormancy-like state in which parasites slow their growth and reduce their metabolic activity, enabling survival during drug exposure without acquiring genetic resistance. Although it remains unexplored whether genetic mutations can alter the frequency of these adaptive drug response states, as observed in fungi, bacteria, and cancer cells (therefore represented by a red question mark before the start of treatment). Both states differ mainly in how broadly the response is expressed across the population. In tolerance, most or all parasites shift from a drug-sensitive state to a moderately drug-tolerant state, allowing the population to survive longer than the wild-type without developing true resistance. In persistence, conversely, only a small subpopulation of persisters enter this state while the remainder of the population remains drug-sensitive. This heterogeneous response leads to the characteristic biphasic killing curve observed in persister populations, reflecting an initial fast killing phase of drug-sensitive cells followed by survival of a persister subpopulation. Upon drug removal, these persisters exit dormancy and resume growth.

Leishmaniasis is a neglected tropical disease (NTD) caused by *Leishmania* parasites, with ~1 million new infections annually worldwide. Despite available chemotherapies, treatment failure and clinical relapse are common. Historically, these outcomes have been attributed to drug resistance, defined by heritable genetic alterations that increase the half-maximal effective concentration (EC_50_) and permit parasite proliferation under drug pressure. However, treatment failure and clinical relapse are not explained by drug resistance alone, as treatment failure may also arise from nonparasite factors such as suboptimal dosing, host immunological status, and pharmacokinetic variability that reduce effective drug exposure [1]. Additionally, drug responses extend beyond classical resistance and include distinct phenomena such as hypersensitivity, reflecting genetic changes that increase drug vulnerability, as well as nongenetic adaptive states such as tolerance and persistence [2], which differ in their behaviour during and after drug exposure. Tolerance describes population-wide prolonged survival under treatment without EC_50_ changes, often linked to metabolic slowdown. Persistence, in contrast, refers to a rare cellular phenotype in which a subpopulation of drug-sensitive cells enters a transient nonproliferative state to survive treatment and subsequently regrows after drug removal, typically producing biphasic killing curves (Fig 1). Both states may partly reflect the proportion of cells entering quiescence, although persistence is not strictly tied to a defined physiological state [2,3]. While persistence is likely intrinsic to *Leishmania* and typically occurs at low-frequency either spontaneously or in response to environmental cues, studies in fungi, bacteria, and cancer cells show that specific mutations can increase entry into such states [4]. Whether similar genetic control exists in *Leishmania* remains unexplored.

Different response states require distinct experimental strategies to uncover underlying mechanisms. Especially defining nongenetic states is critical, as therapies targeting resistance alone may still fail against tolerant or persister populations, sustaining latent parasite reservoirs and relapse syndromes such as post-kala-azar dermal leishmaniasis and leishmaniasis recidivans [5*–*7]. Importantly, dissecting and mapping these diverse responses may enable the design of combination or sequential therapies based on collateral sensitivities, capable of targeting both proliferating parasites and drug-tolerant or persister subpopulations, thereby reducing relapse and treatment failure.

Here, we highlight emerging genomics and perturb-omics approaches in *Leishmania*, emphasising that their impact depends on experimental designs that capture the full range of drug responses.

## What it means to measure the “Full Spectrum” of drug responses

Drug responses are often reduced to EC_50_ shifts, which preferentially capture heritable resistance and hypersensitivity. Resolving tolerance and persistence, however, requires measuring killing kinetics, heterogeneity, and reversibility. Practically, this involves complementing EC_50_ measures with: (i) time-kill curves, (ii) washout/regrowth assays to identify tolerant/persister-like survivors, and (iii) approaches that retain or elucidate population structure, such as barcoding, single-cell profiling, or state-enrichment strategies. Methods must thus capture heritability, heterogeneity, and dynamic state transitions in phenotype during drug exposure.

## Methods that map heritability, heterogeneity, and state

Whole-genome sequencing (WGS) of clinical isolates captures heritable single-nucleotide variants (SNVs), copy number variation (CNV), and aneuploidy associated with drug adaptation. Target-enrichment strategies enable direct sequencing of parasite DNA from patient samples, minimising culture-induced bias [8]. While bulk WGS averages populations and may miss low-frequency variants and mosaic karyotypes, comparisons of cultivated pre- and post-treatment populations can reveal survival without detectable genetic change, consistent with tolerance or persistence. However, such analyses cannot unravel whether variants arise from selection of pre-existing subpopulations or de novo adaptation, particularly in *Leishmania*, where genome plasticity and rapid karyotypic shifts reshape populations over short timescales. Single-cell genome sequencing using semi-permeable hydrogel capsules overcomes this limitation by isolating individual parasites for genome amplification, enabling detection of mosaic aneuploidy and rare variants that may act as reservoirs for adaptation under drug pressure [9]. Longitudinal sampling further distinguishes expansion of resistant sub-clones from survival and rebound of drug-sensitive cells.

Transcriptomics complements genomics by capturing drug-induced expression changes and host context, alongside other systems-level readouts such as proteomics and metabolomics. Bulk RNA-seq defines average response programs [10], whereas single-cell RNA-seq resolves transcriptional heterogeneity and host-parasite state diversity that may influence survival even in the absence of stable resistance genotypes [11]. Chemical genetics provides a complementary route to identify resistance-associated mutations through mutagenesis and sequencing of resistant clones [12].

Collectively, these approaches combine genotype tracking with state-resolved profiling across drug exposure regimens and time-points, enabling discrimination between resistant sub-clone expansion and survival of drug-sensitive cells. Nevertheless, they primarily generate associations and often cannot distinguish causal drivers from passenger variation. This is where perturb-omics becomes transformative.

## Emerging perturb-omics in *Leishmania*: From association to causal networks

Earlier functional studies in *Leishmania* relied primarily on Cos-Seq [13], targeted mutagenesis [14], and limited RNAi-based approaches [15]. Now, gene-editing platforms represent a pivotal expansion. Bar-seq approaches, either barcoding lines with distinct response profiles [16,17] or generating individual barcoded gene deletions using CRISPR [18], enable pooled tracking across drug conditions. Although library construction can be labour-intensive, screens are high-throughput and scalable [19], making bar-seq well suited for multi-condition phenotyping across the spectrum.

Conventional CRISPR-Cas9 knockout screens in *Leishmania* have also succeeded under strong selection for resistance mutations [20]. However, for spectrum mapping, limitations remain: low editing efficiency without stringent selection favours strong resistance determinants, and repair outcomes via microhomology-mediated end joining can be unpredictable.

In contrast, base editing bypasses DNA repair constraints and enables precise nucleotide substitutions. Cas9-based cytosine base editors (CBE) can introduce premature stop codons to generate functional mutations in nonclonal populations [21]. Importantly, base editing also has strong potential for targeted mutagenesis screens, with adenine and dual base editors expanding editable sites [22]. In *Leishmania*, near-genome-wide scalability is achieved by co-expressing an AsCas12a ultra variant and T7 RNA polymerase to integrate sgRNA libraries into a genomic safe-harbour locus [23]. Our recent preprint demonstrated high-throughput loss-of-function screening across 94% of the *L. mexicana* genome [24]. Some limitations remain, including unpredictable effects of truncated proteins or mRNA stability, and the fact that pooled screens infer edits from guide abundance rather than confirming edits at each locus.

An alternative is RNA-targeting CRISPR-Cas13 systems, which degrade transcripts without genome alteration. Cas13b has been established in *Leishmania* [25] and holds strong potential for large-scale drug response screens. But guide design is constrained by RNA secondary structure and collateral RNA degradation, which may affect nearby transcripts upon activation.

**Fig 2:**
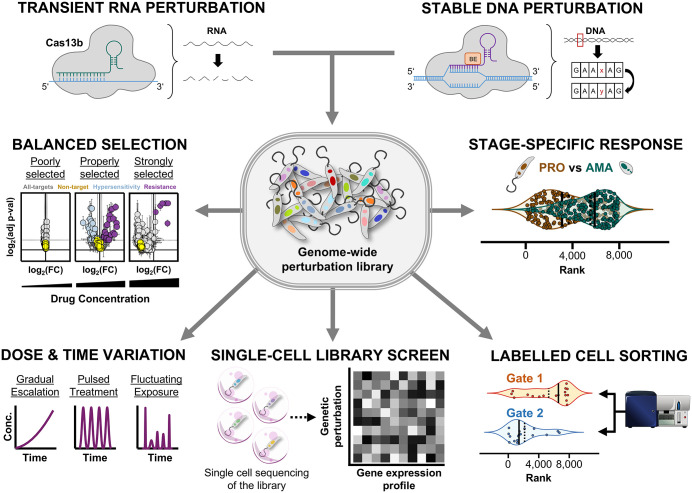
Genome-wide perturbation screens to uncover determinants of antileishmanial drug response beyond classical resistance. Schematic overview of pooled genome-wide perturbation strategies for dissecting diverse parasite responses to drug exposure in *Leishmania*. Perturbation libraries can be generated using transient RNA-targeting approaches (e.g., Cas13b) or stable DNA-editing systems such as CRISPR base editors, enabling systematic interrogation of gene function at the transcript or genomic level. Balanced and calibrated drug-selection pressures are critical for maintaining library representation and identifying relevant determinants, as overly stringent selection may deplete library diversity, whereas weak selection may fail to enrich adaptive variants. Alternative drug exposure regimens, including gradual dose escalation, pulsed treatment, and fluctuating drug exposure, may better model clinically relevant conditions and facilitate identification of nonclassical adaptive phenotypes such as tolerance or persistence. Coupling pooled perturbation screens with single-cell transcriptomics enables resolution of heterogeneous or rare cellular states associated with drug survival. Similarly, fluorescence-activated cell sorting (FACS) of labelled subpopulations can enrich persister-like or stress-responsive parasites for downstream genomic and transcriptomic analyses. Comparative screening across developmental stages, including promastigotes (PRO) and intracellular amastigotes (AMA), may further reveal stage-specific fitness determinants and drug response pathways that are not detectable in a single parasite stage.

Together, these approaches form a complementary toolkit spanning stable genomic disruption and transient transcript-level perturbation, providing a scalable platform to interrogate coding and noncoding genes across the repetitive and copy-number-variable *Leishmania* genome. The optimal perturb-screening strategy depends on the biological question being addressed (Fig 2).

## How to use perturb-omics to move beyond drug resistance-centric models in *Leishmania*

### Calibrated selection pressure to capture the full range

Calibrated drug-selection (using tailored dosing regimens) preserves library diversity and enables detection of both enrichment and depletion signals. This reveals resistance as well as hypersensitivity and collateral sensitivity. In contrast, strong library selection (e.g., a single high-dose of drug) rapidly enriches high-fitness resistance clones, such as miltefosine transporter mutations dominating miltefosine screens [20,24], and masks subtler phenotypes, which often require alternative or state-enrichment strategies to be revealed [24,26].

## Varying dose and exposure time to separate early survival from stable resistance

Applying libraries across multiple drug concentrations and exposure regimens, including gradual dose escalation, pulsed treatment, or fluctuating exposure, enables identification of temporally distinct genetic effects across different stages of drug response during adaptation. Ideally, such experiments should use de novo designed libraries that exclude strong resistance-conferring perturbations, as these can rapidly dominate and obscure more subtle phenotypes. This design distinguishes early survival and tolerance-like perturbations from those driving stable resistance under sustained selection [26].

## Enriching the relevant state to discover persistence determinants

Because persisters are rare, additional strategies are required to uncover contributors to persistence. Selection schemes that eliminate proliferating cells (e.g., suicide switches, which are molecular tools that can be used to induce apoptosis in dividing cells) can enrich noncycling persisters that survive drug exposure in a dormant, nondividing state, prior to genetic or compound screening, revealing vulnerabilities that are largely invisible in bulk populations [27]. Likewise, integrating single-cell state atlases, which capture transcriptional heterogeneity across individual parasites, with perturbation libraries can identify genes regulating entry into tolerance and persistence [28].

## Coupling perturbation libraries to persister-labelling and sorting

Functional screens can be combined with persister-labelling strategies, including proliferation markers (^2^H_2_O incorporation, BrdU/EdU, CFSE dilution) or, more compatible with pooled screens, fluorescent reporters integrated into dormancy-responsive loci [29] that decrease in signal as parasites enter quiescence, enabling FACS-based screens. Emerging methods may also enable positive labelling of quiescent cells using dedicated fluorescent biomarkers, improving selective isolation of dormant subpopulations. Alternatively, photoconvertible reporters such as mKikumeGR allow prospective isolation of persister-like cells [30]. After violet-light exposure, fluorescence shifts from green to red. Metabolically active parasites revert to green fluorescence, whereas persister-like cells remain red due to their nonreplicative, dormant state. When applied under pulsed versus gradual drug exposure, these approaches may reveal pathways regulating tolerance and persister entry, beyond changes in EC_50_.

## Adopting clinically relevant biology

*Leishmania* alternates between promastigotes in sandflies and amastigotes in mammals. Although promastigotes and axenic amastigotes offer the simplest lab-based study system, to better approximate patient-relevant drug responses, screens can be performed in intracellular amastigotes using macrophage and animal models [31]. While these systems provide increasingly physiological contexts, they remain experimental models and do not fully capture the complexity of clinical treatment failure. Finally, integrating resulting datasets into community resources such as TriTrypDB/VEuPathDB enables standardised annotation, meta-analysis, and cross-study re-use [32], supporting the development of predictive models of parasite drug response that extend beyond classical resistance and encompass hypersensitivity, tolerance, and persistence.
